# Rapid Multiorgan Dissemination of Low-Grade Myxofibrosarcoma: A Case Report

**DOI:** 10.1155/2012/310805

**Published:** 2012-09-02

**Authors:** Yasutaka Murahashi, Mitsunori Kaya, Tamotsu Soma, Mikito Sasaki, Satoshi Nagoya, Takuro Wada, Toshihiko Yamashita

**Affiliations:** Department of Orthopaedic Surgery, Sapporo Medical University School of Medicine, S-1, W-16, Chuo-ku, Sapporo, Hokkaido 060-8543, Japan

## Abstract

Myxofibrosarcoma is one of the most common sarcomas in the extremities of elderly people. It is characterized by a high frequency of local recurrence due to an infiltrative growth property. In contrast, the overall risk of distant metastases is generally low. This makes the prognosis for the patients with myxofibrosarcoma definitely good. In this paper, we will report the case of a 79-year-old female with very aggressive metastatic low-grade myxofibrosarcoma. The disease progression was really unexpected and misled every possible medical interpretation, leading to rapid worsening of the patient's clinical conditions and no chance for therapy. The tumor developed diffuse infiltration in lung, spine, skeletal bone, abdomen, paravertebral muscles, and liver. The patient died 8 months after the diagnosis of remote metastases due to rapid tumor progression.

## 1. Introduction

 Myxofibrosarcoma is the most common soft tissue sarcoma in the extremities of older adults [1]. Although it has a high rate of local recurrence, the overall risk of distant metastases is relatively low compared to other sarcoma subtypes [1]. In this paper, we described a case of multiorgan metastatic myxofibrosarcoma with very aggressive behavior. 

## 2. Case Presentation

**Figure 1:**
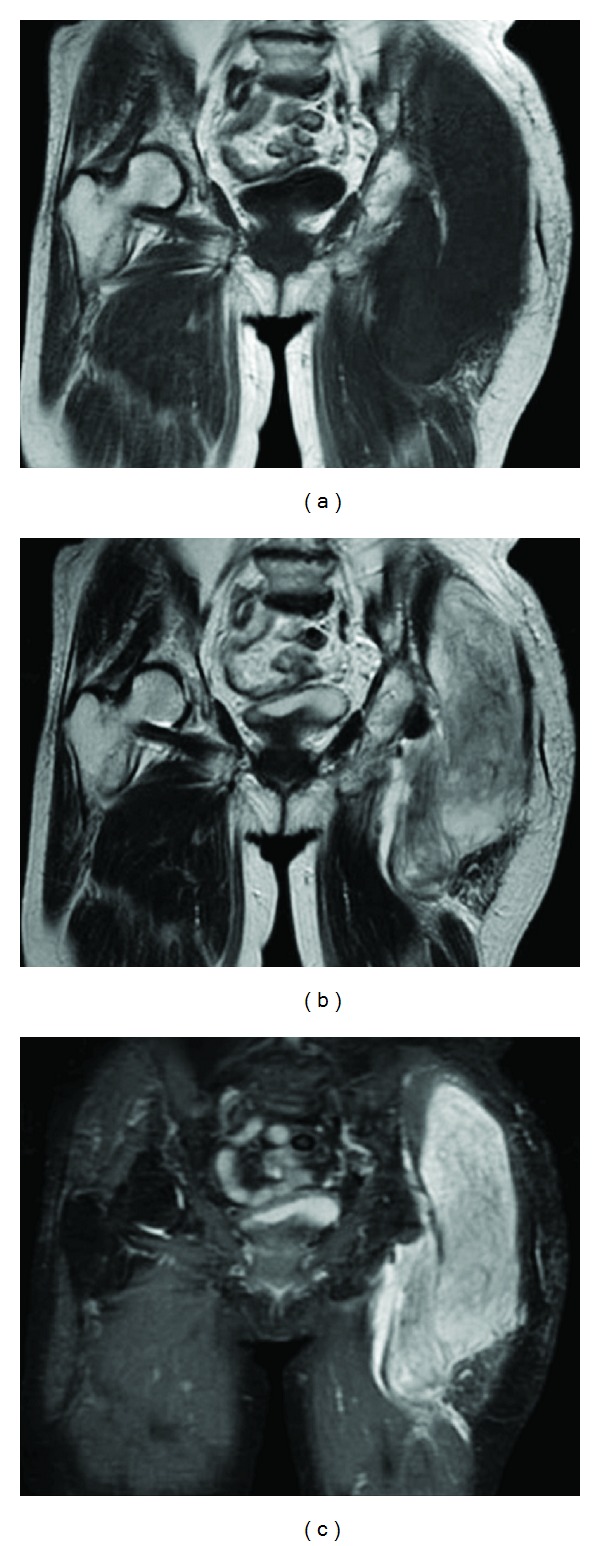
Magnetic resonance images of the left buttock. (a) T1-weighted, (b) T2-weighted, and (c) T2-STIR MR images demonstrated a multilobular intermuscular mass.

**Figure 2:**
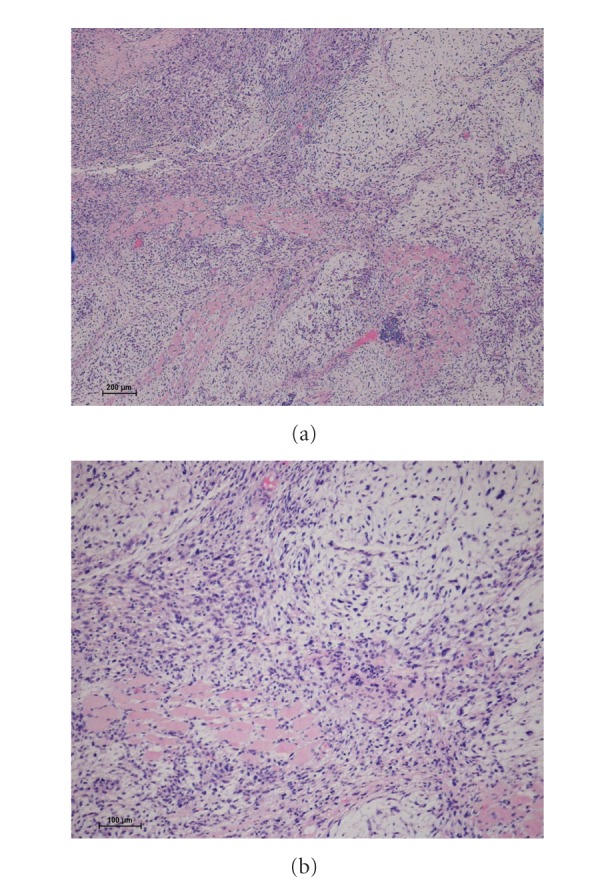
Hematoxylin and eosin stain ×40 (a) and ×100 (b). Biopsy of the tumor showed a typical histology of myxofibrosarcoma. The histological grade was determined as grade 1.

**Figure 3:**
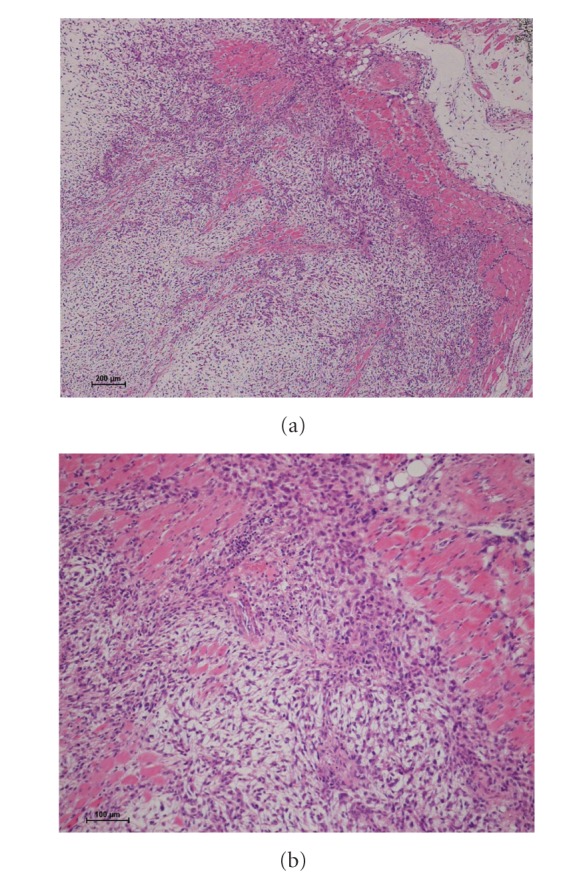
Hematoxylin and eosin stain of the resected tumor (a) ×40 and (b) ×100. Spindle-shaped tumor cells were arranged within the loose myxoid stroma. Only a few atypical mitoses and necroses were found. The histological grade was determined as grade 1.

**Figure 4:**
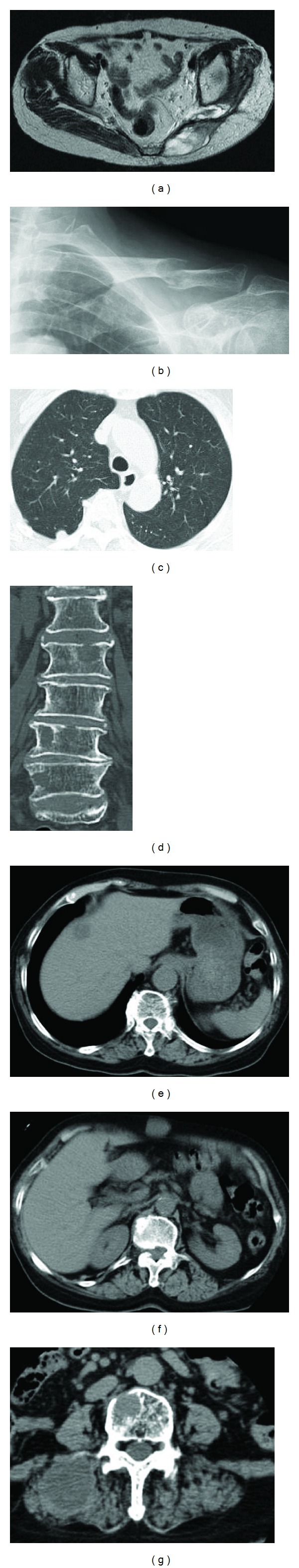
15 months after the operation, MR images, plain X-ray, and computed tomography demonstrated (a) a local recurrence and multiorgan dissemination including the (b) clavicle, (c) lung, (d) spine, (e) liver, (f) abdomen, and (g) paravertebral muscles.

**Figure 5:**
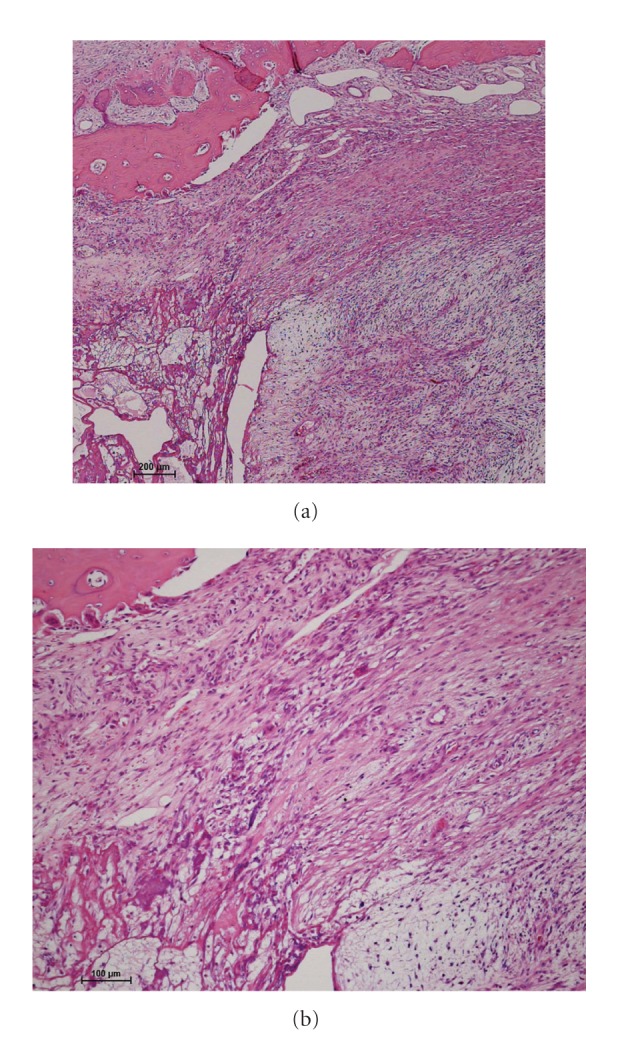
Hematoxylin and eosin stain of metastatic lesion of clavicle (a) ×40 and (b) ×100. The histological grade was also determined as grade 1.

 A 79-year-old female presented to our institution with a lump on her left buttock that had appeared one month previously. Physical examination revealed a dome-shaped tumor, 22 cm × 14 cm in diameter. The tumor was elastic, hard, and asymptomatic. No regional lymph nodes were palpable. Magnetic resonance imaging (MRI) demonstrated a multilobular intermuscular mass on the left buttock (Figures 1(a)–1(c)). The tumor was attached to the proximal femur, and the sciatic nerve was also involved. Abnormal signal extension along the muscle fascia of the gluteal muscles was obvious. An excisional biopsy showed spindle-shaped tumor cells in the loose myxoid stroma. The nuclei of the tumor cells were slightly hyperchromatic and irregularly shaped. Only a few atypical mitosis and necrosis were found. Based on the histopathological features, the tumor was diagnosed as myxofibrosarcoma (Figures 2(a) and 2(b)). To determine the histological grade, the FNCLCC tumor grade was assigned using a modified updated version of the original FNCLCC system based on three criteria: tumor differentiation, the mitotic index, and tumor necrosis [1]. A score was attributed independently to each parameter, and the grade was obtained by adding the three attributed scores. Lesions with a total score of 2 or 3 were classified as grade 1, whereas total scores of 4 or 5 and 6 were deemed grade 2 and grade 3, respectively [1]. In this case, the histological grade was determined as grade 1. As the tumor was attached to the proximal femur and the sciatic nerve, hip disarticulation was necessary to achieve a tumor-free margin. However, we speculated that postoperative radiation therapy with limb-sparing surgery could prevent the local recurrence of an extremely low-grade myxofibrosarcoma. In addition, we hesitated as such a radical procedure would lead to serious functional loss. Therefore, we performed limb-sparing surgery. The tumor was resected with the gluteal medius muscle, the gluteal maximum muscle, and the adductor muscle. The tumor was stripped from the proximal femur. The sciatic nerve was preserved by removing it from the tumor. As the tumor was exposed intraoperatively, the surgical margin was determined as grossly positive. The histological grade of the resected tumor was also determined as grade 1 (Figures 3(a) and 3(b)). To prevent local recurrence, 60 Gy of postoperative radiation was delivered. The postoperative period was uneventful; however, 15 months after the operation, an MRI showed local recurrence (Figure 4(a)). A plain X-ray taken for the evaluation of her left shoulder pain showed a pathological fracture and metastatic bone tumor of the left clavicle (Figure 4(b)). A total body computed tomography scan documented multiorgan dissemination including the skeletal bones, lung, whole spine, liver, subcutaneous layer of abdomen, and paravertebral muscles (Figures 4(b)–4(g)). To control the pain due to a pathological fracture of the clavicle, a partial claviculectomy was performed. Histological grading was still grade 1 (Figures 5(a) and 5(b)). There was no progression to a high-grade lesion. To prevent spinal cord injury due to a pathological fracture of the spine, radiation therapy was delivered to the whole spine. However, the patient's serious physical status did not permit further chemotherapy, and she died 8 months after diagnosis of remote metastases due to rapid tumor progression.

## 3. Discussion

 Myxofibrosarcoma is one of the most common sarcomas in the elderly. It was first described in 1977, and it represents a spectrum of myxoid fibroblastic sarcomas, the high-grade end of the spectrum of what was formerly labeled as “myxoid malignant fibrohistiocytoma” [2–4]. Currently, the World Health Organization recognizes myxofibrosarcoma as a distinctive entity, marked by distinctive clinicopathologic characteristics [1, 5]. Compared to other sarcoma subtypes, the prognosis of myxofibrosarcoma is relatively good, with an overall risk of metastases ranging between 20% and 25% [4–7].

 In the present case, spindle-shaped tumor cells were sparsely distributed in the abundant myxoid matrix. The cellularity was remarkably low, and tumor necrosis was not obvious. From these findings, the present case was diagnosed as grade 1 myxofibrosarcoma. Tumor grades, as in the most soft tissue sarcoma subtype, predict the risk of developing distant metastases. Generally, grade 1 cases have been considered to be incapable of metastatic disease [6, 8, 9]. Most of the patients who eventually developed metastatic disease reported in the several literatures had grade 2 or 3 myxofibrosarcoma [6, 8, 9]. Within our knowledge, only a few cases with grade 1 myxofibrosarcoma including the current case developed metastatic lesions [10]. 

 The diameter of the tumor was also a risk factor for the establishment of distant metastases [9–11]. In the current case with grade 1 myxofibrosarcoma, we decided to perform the planned inadequate resection to reduce the functional disability and to spare the lower limb. For the prevention of local recurrence, we delivered postoperative radiation therapy. The current case had a deep-seated large tumor sized more than 20 cm in diameter, and therefore, the current case still had a high risk of the development of metastatic lesions. As a result of the limb sparing surgery, rapid development of local recurrence and multiorgan dissemination had developed. More radical surgical management including amputation was mandatory even for the current case with a grade 1 myxofibrosarcoma. 

 The present case was remarkably hypocellular and only slightly pleomorphic in histology that made us diagnose grade 1 myxofibrosarcoma. However, the patient developed fatal metastases in multiple vital organs. These facts remind us that myxofibrosarcoma can grow and spread rapidly even if its malignant histopathological grade is extremely low. Our observation suggests that the strict managements of the primary tumors are necessary even for patients with low-grade myxofibrosarcoma, especially in the cases located in the deep layer with extremely large size.
